# Performance Evaluation of LoRa Considering Scenario Conditions

**DOI:** 10.3390/s18030772

**Published:** 2018-03-03

**Authors:** Ramon Sanchez-Iborra, Jesus Sanchez-Gomez, Juan Ballesta-Viñas, Maria-Dolores Cano, Antonio F. Skarmeta

**Affiliations:** 1 Department of Information and Communication Engineering, University of Murcia, 30100 Murcia, Spain; jesus.sanchez4@um.es (J.S.-G.); skarmeta@um.es (A.F.S.); 2 Department of Information and Communication Technologies, Universidad Politecnica de Cartagena, 30202 Cartagena, Spain; juanballesta1992@gmail.com (J.B.-V.); mdolores.cano@upct.es (M.-D.C.)

**Keywords:** LP-WAN, IoT, Smart cities, LoRa, LoRaWAN

## Abstract

New verticals within the Internet of Things (IoT) paradigm such as smart cities, smart farming, or goods monitoring, among many others, are demanding strong requirements to the Radio Access Network (RAN) in terms of coverage, end-node’s power consumption, and scalability. The technologies employed so far to provide IoT scenarios with connectivity, e.g., wireless sensor network and cellular technologies, are not able to simultaneously cope with these three requirements. Thus, a novel solution known as Low Power - Wide Area Network (LP-WAN) has emerged as a promising alternative to provide with low-cost and low-power-consumption connectivity to end-nodes spread in a wide area. Concretely, the Long-Range Wide Area Network (LoRaWAN) technology is one of the LP-WAN platforms that is receiving greater attention from both the industry and the academia. For that reason, in this work, a comprehensive performance evaluation of LoRaWAN under different environmental conditions is presented. The results are obtained from three real scenarios, namely, urban, suburban, and rural, considering both dynamic and static conditions, hence a discussion about the most proper LoRaWAN physical-layer configuration for each scenario is provided. Besides, a theoretical coverage study is also conducted by the use of a radio planning tool considering topographic maps and a precise propagation model. From the attained results, it can be concluded that it is necessary to evaluate the propagation conditions of the deployment scenario prior to the system implantation in order to reach a compromise between the robustness of the network and the transmission data-rate.

## 1. Introduction

Low Power - Wide Area Networks (LP-WAN) have emerged as one of the most promising Internet of Things (IoT) enabling technologies due to their advantages over other solutions such as short-range transmission technologies (e.g., WiFi, Bluetooth, IEEE 802.15.4-based protocols, etc.) or cellular communications [1,2]. This fact is motivated by the different requirements that current and future IoT systems present in comparison with traditional Wireless Sensor Networks (WSN) or cellular systems. These conceptual differences between the IoT, WSN, and cellular-network paradigms are presented in Table 1. Observe that, basically, the main characteristics and requirements that an IoT system presents are: (i) a reduced use of bandwidth; (ii) a limited number of messages per node per day; (iii) huge number of simultaneously-connected end-devices; (iv) long-range links; and (v) low-cost end-devices. The majority of these features are satisfied by the different LP-WAN platforms in the market [3]; however, they present important differences mostly related to their network architecture and the adopted business model. In this work, the focus is on LoRaWAN (Long-Range Wide Area Network) [4], which is a platform promoted by the LoRa Alliance (IBM, Semtech, and Cisco, among others), positioned together with Sigfox [5] at the top of the rank of platforms receiving more attention from both the academia and the industry [1]. Although both of them offer similar features to their users, each one takes different technological approaches. On the one hand, Sigfox presents a highly closed and limited transmission scheme with very few adaptation capabilities as it employs a proprietary ultra-narrow band technology with a maximum uplink data-rate of 100 bps and a maximum packet-payload of 12 Bytes. As the majority of the LP-WAN platforms, Sigfox uses the Industrial, Scientific, and Medical (ISM) sub-GHz frequency bands so, due to the strict duty-cycle limitations imposed by worldwide regulations, the number of transmissions is highly restricted; for example, in Europe, a maximum of 140 messages per end-node per day are allowed. Nevertheless, despite these technological limitations, Sigfox is having great success due to its business and architecture model that consists of a wide network of base-stations covering great part of some European territories and a backhaul network that permits to deliver the collected data from the edge-network to the cloud almost with reduced latency. Therefore, the end-users are only in charge of acquiring and installing the end-devices, because data-transport and its presentation is in Sigfox hands, which notably eases the management tasks from the user side.

**Figure 1:**
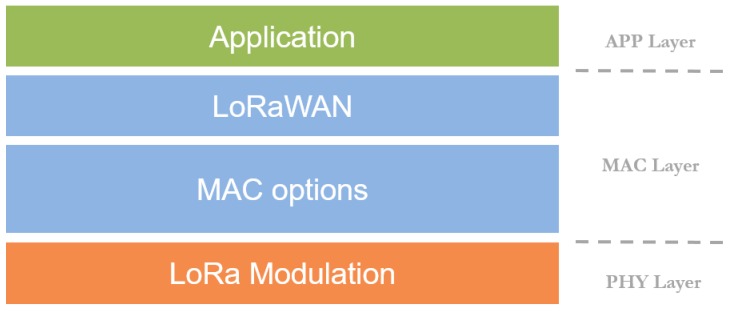
Long Range Wide Area Network (LoRaWAN) stack.

In turn, LoRaWAN [4] presents a more advanced solution that permits the end-user to adapt the system to her needs. Specifically, LoRaWAN defines the OSI’s (Open Systems Interconnection) Physical (PHY) and Medium Access Control (MAC) layers (please, see Figure 1). In the PHY layer, LoRaWAN employs their own-developed modulation scheme, so-called LoRa (Long Range), which is a proprietary technology based on a Chirp Spread Spectrum (CSS) modulation that permits low-power long-range transmissions. On the MAC layer, LoRaWAN defines the communication protocol, the system architecture, and other services and interfaces for higher-layer applications. Both layers present some customizable settings that provide the system with a valuable flexibility to adapt it to different use cases. Further insights about LoRa and LoRaWAN are provided in subsequent sections.

The main motivation of this work is characterizing LoRaWAN PHY layer features in order to be able to select the most proper configuration depending on the conditions of the deployment scenario. Concretely, the focus is on a mobile scenario in which the end-node is placed onboard a vehicle; these conditions are similar to those of services that have been lastly associated to this type of radio technologies, such as fleet tracking, goods monitoring, or vehicle state control, among others [6,7]. As a first step, a theoretical coverage study is presented by means of the expected power-level maps obtained from an accurate radio-network planning tool [8], which makes use of topographic maps and the widely-used Okumura–Hata propagation model. Thereafter, this coverage study is validated by comparing its predictions with the outcomes attained in an experimental sampling campaign. Previous works have demonstrated the need of contrasting the predictions of IoT network planning tools with the results extracted from real experiments [9]. Therefore, in the following sections, a comprehensive exploration of LoRaWAN is conducted by examining its performance under different configurations in different mobile scenarios, namely, urban, suburban, and rural. With the aim of completing this study, in addition to this evaluation under mobility conditions, a nomadic test has been also conducted in the same scenarios in order to find performance differences between them.

The main contributions of this work are the following:

- An exhaustive evaluation of LoRaWAN with emphasis in its adaptation capabilities is presented.
- The performance of LoRaWAN in different environments with pre-defined propagation conditions, namely, urban, suburban, and rural is explored. The impact of end-node mobility on the system performance is also discussed.
- Under this diversity of scenarios, the best LoRaWAN configuration for each use case is identified and discussed.

The rest of the paper is organized as follows. In Section 2, a review of the most prominent works regarding the evaluation of LoRaWAN deployments is presented. Section 3 introduces the main foundations of LoRa and LoRaWAN. The methodology, tools, and equipment used in this work are described in Section 4. Section 5 presents and discusses the results obtained from both the theoretical coverage study and the experimental campaign. Finally, Section 6 concludes this work, presenting the most important findings.

## 2. Related Work 

Although the appearance and widespread of LoRaWAN are recent, a number of works have been published aiming at analyzing or evaluating its performance in different scenarios or proposing enhancements to the off-the-shelf version of LoRaWAN [7,10,11,12,13,14,15,16,17,18,19]. From a theoretical perspective, works in [10,11,12] analyzed the capacity of LoRaWAN in terms of scalability and node-throughput. All these works concluded that LoRaWAN systems should be carefully configured and dimensioned with the aim of hosting a great number of end-devices. Concretely, in [10], the negative impact of interferences within highly-populated LoRaWAN cells was studied. Authors found some issues related to the co-spreading sequence interference, which notably harms the scalability of LoRaWAN systems. However, as stated in [11], LoRaWAN networks can be generally utilized for fairly dense deployments with relaxed latency or reliability requirements. Thus, in order to ensure the network scalability when the end-device population prominently grows, two measures can be taken: (i) the number of delivered packets per node per day should be reduced; or (ii) the number of gateways should be increased [12].

As mentioned above, some works have proposed enhancements to the original LoRaWAN features [13,14]. In [13], authors presented a solution to improve the overall security of a LoRaWAN-based IoT system. This proposal employed proxy-nodes for performing the cryptographic operations in order to avoid heavy computation in the constrained end-nodes. In turn, work in [14] presented an integration of IPv6 into LoRaWAN. Similar to the case of 6LoWPAN, this solution permitted a higher interoperability of the IoT network with the outside world. Although interesting, both works lacked of a detailed performance evaluation to demonstrate the impact of their proposals on the system operation.

From a different perspective, other studies presented the results extracted from experimental tests conducted in diverse scenarios and situations [15,16,17,18,19]. These works focused on evaluating the performance of LoRaWAN under different propagation and environmental conditions. For example, in [15], a real LoRaWAN deployment was evaluated and it was found that the base-station antenna location and elevation have great importance in the network performance. The measurements were carried out using three different base-stations. A similar experimental study was elaborated in [16]. In this case, authors focused on tuning the LoRaWAN PHY layer, i.e., LoRa, configuration parameters, identifying both Spreading Factor (SF), which will be explained later in detail, and data-rate as the principal factors impacting on the network coverage. Another coverage study focused on LoRa, was presented in [17], but, in this case it was carried out in an indoor scenario. The presented outcomes demonstrated the robustness of LoRa in adverse industrial environments, even with high data-rates. In turn, authors of [18] investigated the coverage of LoRaWAN in different environments by placing the end-device onboard a car and a boat. Interesting coverage ranges over 10 km were reached with a not excessive Packet Loss Rate (PLR). A more elaborated work was developed in [19], in which the same authors extended their measurements and evaluated the performance of the system under mobility conditions. From a simulation perspective, the work in [7] focused on vehicular scenarios and examined the performance of LoRaWAN in vehicular opportunistic networks, showing better results in comparison with WiFi technology.

As observed in the reviewed works, the coverage range and the performance of different LoRaWAN configurations were evaluated. However, these studies did not characterize the sampling points depending on their adversity against wireless transmissions. In this work, we evaluate the performance of LoRaWAN under three well-defined conditions, namely, urban, suburban, and rural scenarios. By using this methodology, we identify the most proper configuration for LoRaWAN PHY layer parameters in order to reach the best performance in each type of scenario. Besides, a comparison of the attained experimental results with a theoretical propagation model is also presented. A similar approximation was considered in [18] but, in this work, the attained coverage range was compared with the predictions given by the simple Free-Space model. This model is known to be inadequate to predict path loss in complex scenarios like those with the presence of obstacles. For that reason, in the present work, we make use of the predictions provided by a network-planning tool employing the widely used Okumura–Hata propagation model over realistic topographic maps.

## 3. LoRaWAN

As explained in previous sections, the LoRaWAN framework defines two well-differentiated layers, the PHY layer, defined by the LoRa modulation, and the MAC layer, defined by the LoRaWAN protocol. Regarding the former, LoRa is a proprietary spread spectrum modulation scheme that derives from Chirp Spread Spectrum (CSS) modulation that focuses on providing robustness to long-range transmissions. LoRa presents three different configurable parameters, namely, Spreading Factor (SF), Coding Rate (CR), and Bandwidth (BW). By tuning these factors, some of the end-to-end communication features can be adjusted, e.g., data-rate, error-correction capability, and transmission range, among others. Regarding the SF, the spread spectrum modulation is performed by representing each bit of information by multiple chips [4]. Thus, the SF represents the ratio between the chip rate and the baseband information rate. Usual SF values range from 7 to 12, so greater SF values increase the robustness of the transmission link by incrementing the receiver-equipment sensitivity at the expense of the transmission data-rate. On the other hand, by decreasing the SF, the data-rate is notably increased but the transmitted signal needs to be received at higher power-level for its properly decoding. Note that a complementary notation to the SF is the so-called Data Rate (DR); in this case, the scale is reversed, so DR0 is equivalent to SF12, and DR5 is equivalent to SF7. From now on, the term DR will be used. With the aim of additionally improving the robustness of the link, LoRa employs cyclic error coding to perform forward error detection and correction. Such error coding incurs a transmission overhead (extra bits in the LoRa PHY layer payload) that is determined by the CR parameter, which can be set to the following values, 4/5, 4/6, 4/7, and 4/8. Finally, the most employed BW in LoRa transmission is 125 kHz, although bandwidths of 250 kHz and 500 kHz are also supported. More details about LoRa can be found in [4].

**Figure 2:**
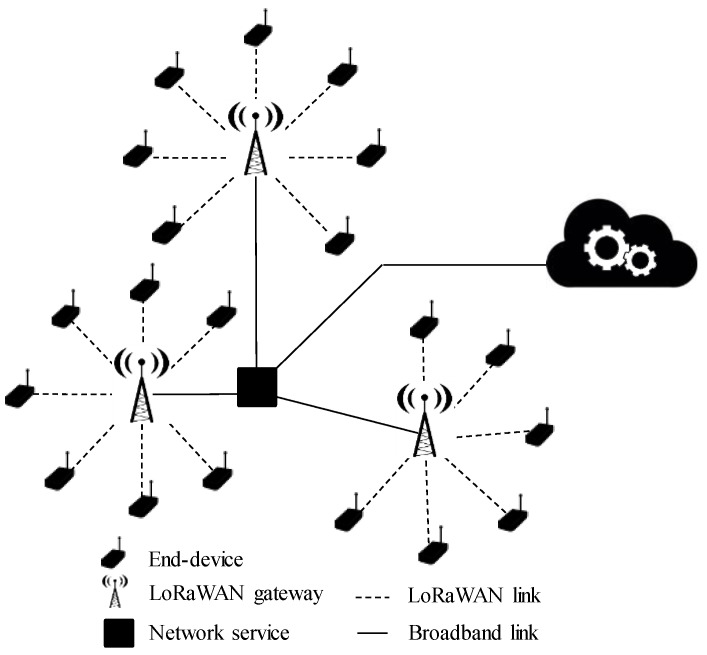
LoRaWAN star-of-stars topology.

From a higher-layer perspective, LoRaWAN employs a star-of-stars topology that permits end-nodes reaching their corresponding gateway via a direct link (see Figure 2). Due to the energy-related constraints of end-nodes, LP-WAN technologies present a highly limited downlink (transmissions from the base-station down to end-nodes), hence LoRaWAN contemplates the use of three different types of end-devices according to their energy limitations and downloading needs: Class A devices are just able to receive transmissions after each uplink connection by opening a listening window. Class B devices are able to accept downlink transmissions during additional scheduled downlink windows. Finally, Class C devices are continuously listening to the channel so they can receive downlink connections at any time. The most employed devices, Class A, present an optimized battery duration of about 5 years [4]. In its regular use, LoRaWAN makes use of the ISM bands, although it can be configured to support licensed spectrum as well. Thereby, LoRaWAN is capable of demodulate signals received 19.5 dB below the noise floor. This allows to achievie very long transmission distances far from those reached by the usual cellular technologies. Depending on the configuration of PHY layer parameters, the data-rate ranges from 0.25 kbps (0.98 kbps in North America due to FCC (Federal Communications Commission) limitations) up to 50 kbps. The maximum permitted payload length is 242 Bytes, which although limited, may satisfy the demands of certain services. Finally, LoRaWAN also considers security issues, so a cryptographic suite based on AES encryption is included by default in order to secure the transmitted data at different OSI levels.

## 4. Material and Methods

As stated above, the performance of LoRaWAN PHY layer has been tested in different scenarios characterized by their level of adversity against wireless transmissions. Recall that the configurable parameters of LoRaWAN PHY layer when using the LoRa modulation are SF, CR, and BW. Note that the GFSK (Gaussian Frequency Shift Keying) modulation, also available in the LoRaWAN specification, has not been considered in this work as it has demonstrated poorer performance in comparison with LoRa [2]. These tunable parameters affect the data-rate and the Packet Delivery Rate (PDR) of the transmissions as showed in the cited previous study [2]. In our study, the possible values that were assigned to the different parameters under consideration were: (i) SF: 7 to 12; (ii) CR: 4/5 and 4/8; and (iii) application payload size: 20 and 40 bytes. 

LoRaWAN transmissions make use of the unlicensed ISM bands, so the European LoRaWAN end-devices must comply with the ETSI 300 220 regulation. This regulation enforces a duty-cycle policy on channels for every sub-band. The LoRaWAN default channels, namely, 868.1, 868.3, and 868.5 MHz use a BW of 125 kHz. Besides, other channels can be manually configured by the user, hence for our tests, the 867.1, 867.3, 867.5, 867.7, and 867.9 MHz channels were employed because they were free of other transmissions during the sampling campaign. The channel employed by the end-device was randomly selected for each transmission. 

**Figure 3:**
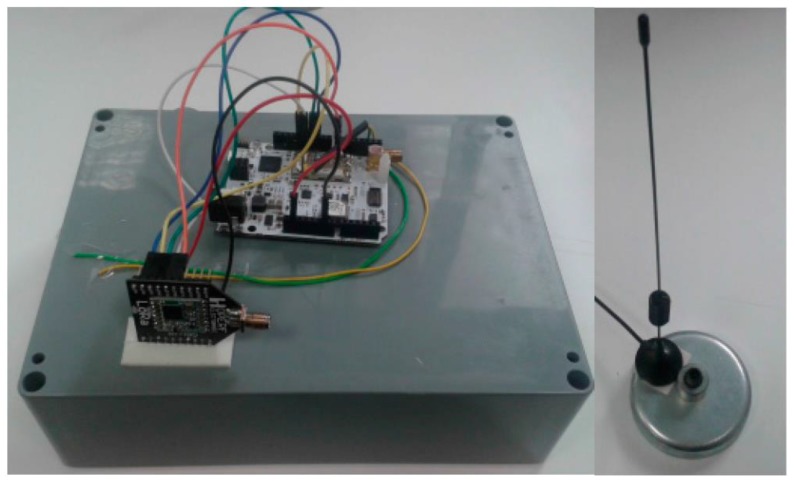
On-board unit detail.

**Figure 4:**
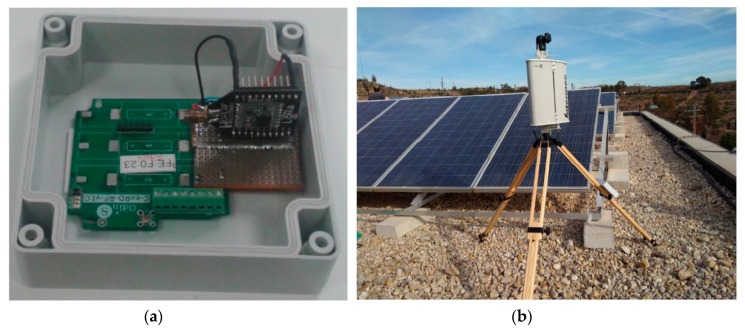
Base-station detail. (**a**) Controller; (**b**) Antenna.

In order to perform the field tests, following the architecture adopted in our previous work [2], the LoRaWAN end-node was placed on the roof of a car, forming part of an On-Board Unit (OBU) that directly communicated with the LoRaWAN base-station. The microcontroller used in the OBU was the Arduino-compatible SmartEverything Fox board by Arrow. Connected to that board, the LoRaWAN radio module was the RN2483 chip by Microchip with an omnidirectional 5 dBi-gain antenna for end-devices, which was kept pointing upwards, favoring the reception gain (see Figure 3). The transceiver chip was a Class A LoRaWAN device with an output power up to 14 dBm. Thus, observe that only LoRaWAN communications were enabled in the OBU. The OBU microcontroller board had an embedded Global Positioning System (GPS) chip that the device used to obtain its location. In addition to the end-device, the other communication end-point, i.e., the base-station, employed the RHF2S008 board by RisingHF, which includes a Semtech SX1301 chip. This module was capable of decoding up to 8 different receptions in different channels employing any SF, simultaneously. Considering these rich features, only one base-station was used in the experimental tests; a sectorial antenna of 8 dBi gain and a far-field beam-width of 65 degrees was attached to the processing board (see Figure 4). A summary of the equipment configuration is shown in Table 2. The base-station relayed the packets back and forth to the LoRaWAN network server, hence the base-station acted as a gateway between the controller and the end-device. In turn, the network server managed the end-device login and acknowledgement (ACK) logics defined in the LoRaWAN specification [4].

Besides, the network server also ruled the downlink queue for managing the different higher-layer packets and it stored the frames received from the OBU as well.

Now that the hardware setup has been described, next the behavior of the involved devices is presented. The OBU acted as a periodic sender hence no other elements or sensors beyond the strictly necessary were involved in the tests. The overall functioning was as follows. First, the OBU obtained its GPS location and transmitted a packet with the coordinates included in the payload field. After transmitting each packet, the OBU waited for an ACK from the base-station using the first receiving window specified in the LoRaWAN protocol (please, see [4]). For each transmission, a different set of the LoRaWAN configuration parameters, i.e., SF, CR, and BW, was employed for both up and down links. This process was periodically repeated during the duration of the test in an infinite loop as described in [20]. This strategy permitted to test different LoRa configurations simultaneously in the different scenarios under consideration. In this procedure, even if the OBU did not receive the ACK after a packet transmission, it kept running, sending a new packet after a given time-out period. The base-station continuously listened to packets and answered with a LoRaWAN ACK to each received packet. Furthermore, the base-station was configured to only answer our OBU packets, discarding in the network server side any other possible LoRaWAN received transmissions in order to avoid extra interferences or unexpected transmissions. Note that only the LoRaWAN packets transmitted from the OBU were detected by the base-station during the tests. The equipment logs showed no transmissions from other devices.

As aforementioned, the tests were carried out on three different scenarios, namely, urban, suburban, and rural, which were selected depending on their architectural configuration. In all the scenarios, the same equipment and software described above was used. The OBU was placed on the vehicle, which circulated in selected routes to perform the tests. As we were interested on evaluating a realistic dynamic scenario, the vehicle speed was affected by real traffic conditions. The maximum, minimum, and average speeds, including confidence intervals (α = 0.05), are presented in Table 3. The routes were the same for all the studied LoRaWAN PHY layer settings, so the results can be analyzed in a comparatively way for each considered scenario. The urban one was located in the city of Murcia, Spain, while traveling through its downtown. The base-station was placed on the Faculty of Computer Sciences in the University of Murcia. The base-station placement was at the edge of the rooftop of a five-story building located in an elevated position and its antenna was pointed towards the test-area by studying its radiation pattern, the distance to the sampling area, and making use of the radiation map obtained from the theoretical coverage study. This process was done trying to optimize the overall reception gain of each experiment, so the antenna remained untouched during the duration of each test. The followed route during the test included the main downtown streets with up to three lanes and high buildings and constructions of up to 15 stories in the surroundings. In turn, the suburban scenario had less adverse conditions for the radio communications, with isolated shorter buildings of up to two stories and some vegetation areas. For this scenario, the base-station placement was at the same point as in the urban one, but the antenna was accordingly pointed to the area of study. The route traveled through industrial and residential areas, with some gentle terrain elevation changes. Finally, the rural scenario was the most favorable as it presented almost free-space conditions. The terrain in which the tests were performed was flat, with lack of large constructions and barely high-vegetation. In this case, the base-station was placed in the rooftop of the Technology Transfer Center in the Fuente Álamo Technology Park, a 4 -story building. The selected route traveled up to 20 km east from the location of the base-station through low transited roads.

As aforementioned, prior to our experimental study, we have examined the expected signal level in the afore-described scenarios by using the well-known Okumura–Hata propagation model. To this end, an accurate radio planning tool including topographic maps and high grade of flexibility that permits introducing the characteristics of the employed equipment has been used [8]. The Okumura-Hata model is an extensively used propagation model for planning wide-area deployments. It covers a wide range of frequencies and types of services, so it is necessary to properly adjust the equipment features such as receivers sensitivity, antennas type, transmission power, carrier frequency, etc. (please, see Table 2).

## 5. Results and Discussion

In this Section, the outcomes obtained in the different scenarios under study are presented. As explained above, different LoRaWAN PHY layer configurations have been tested in three environments that are discriminated by their particular conditions against wireless transmissions. This adversity is determined by the number and density of obstacles presented by each of them. Thus, the following scenarios have been considered: (i) urban: high adversity against wireless conditions; (ii) suburban: medium adversity against wireless conditions; and (iii) rural: low adversity against wireless conditions. As stated above, the study has been divided into two well-differentiated stages. First, the theoretical results calculated by a radio planning tool are shown; thereafter, these results are compared by those extracted from the experimental sampling campaign. 

### 5.1. Theoretical Coverage Study

**Figure 5:**
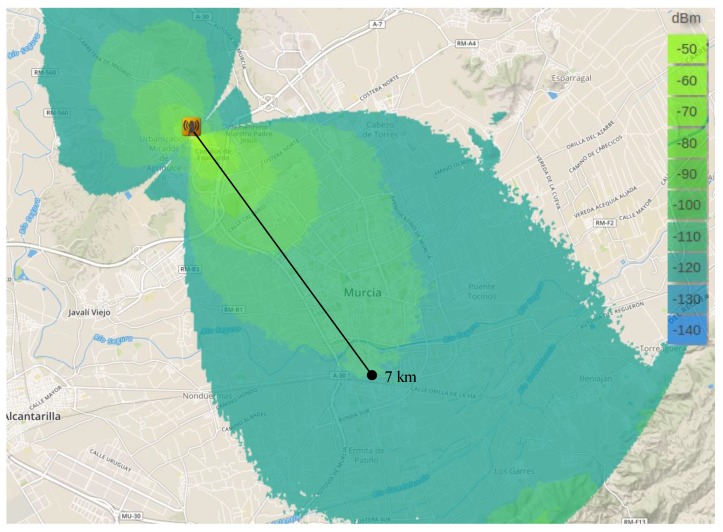
Theoretical coverage map by considering the Okumura–Hata propagation model. Urban scenario.

**Figure 6:**
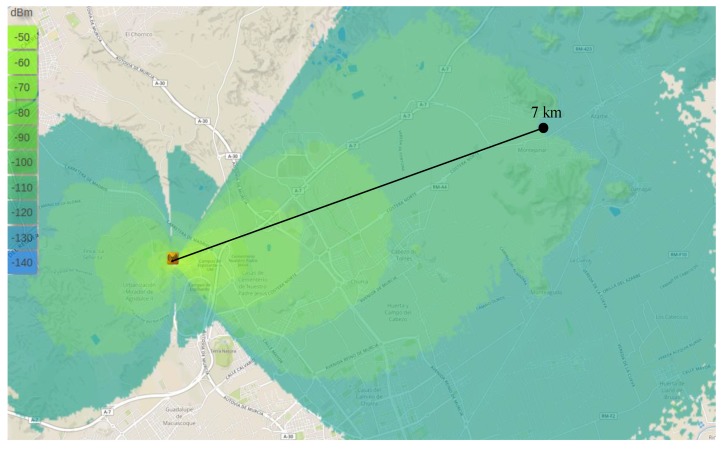
Theoretical coverage map by considering the Okumura–Hata propagation model. Suburban scenario.

**Figure 7:**
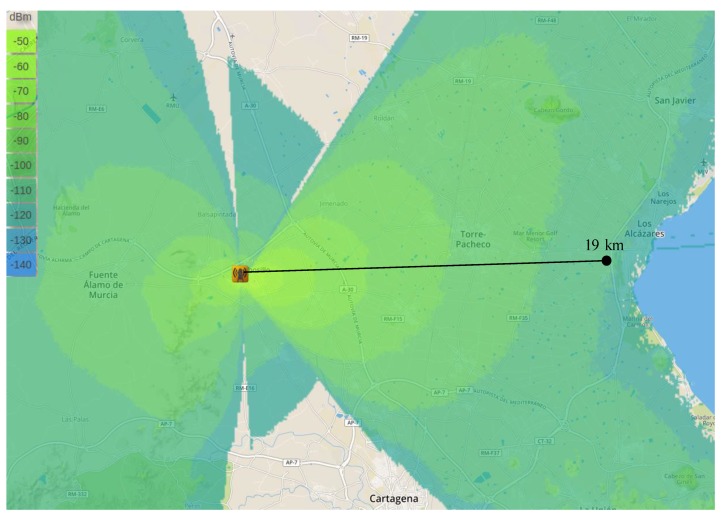
Theoretical coverage map by considering the Okumura–Hata propagation model. Rural scenario.

As aforementioned, before performing the experimental study, we used a radio planning tool [8] that permited us to estimate the signal level within the areas covered by the base-station in the different scenarios. This tool employs topographic maps in order to consider the impact of the terrain elevations on the signal propagation and presents highly configurable options to precissely simulate the characteristics and conditions of the real equipment. Thus, by configuring this tool with the features corresponding to our test-bed configuration, i.e., antennas’ gain and height, receiver sensitivity, transmission power, etc. (Table 2), the coverage maps showed in Figure 5, Figure 6 and Figure 7 have been obtained. Recall that the propagation model applied for obtaining these estimations is the Okumura-Hata model. Observe that the green areas represent the levels of received power above the card sensitivity at the lowest DR, i.e., ≈−130 dBm [4]; for the sake of clarity, we have indicated the length of these theoretical maximum link distances. In turn, the blue areas represent a level of received power that is below the receiver’s floor sensitivity. Thus, observe that for both, the urban and suburban scenarios the planning tool predicted a link range about 7 km, while in the rural environment 19 km was the maximum predicted distance. The similar predictions for the two first scenarios can be explained by the presence of obstacles in both of them, which limited the maximum reached range; in turn, the complete absence of obstacles in the rural scenario permitted a notable maximum range that is not usually reachable by traditional IoT communication technologies.

### 5.2. Experimental Results

**Figure 8:**
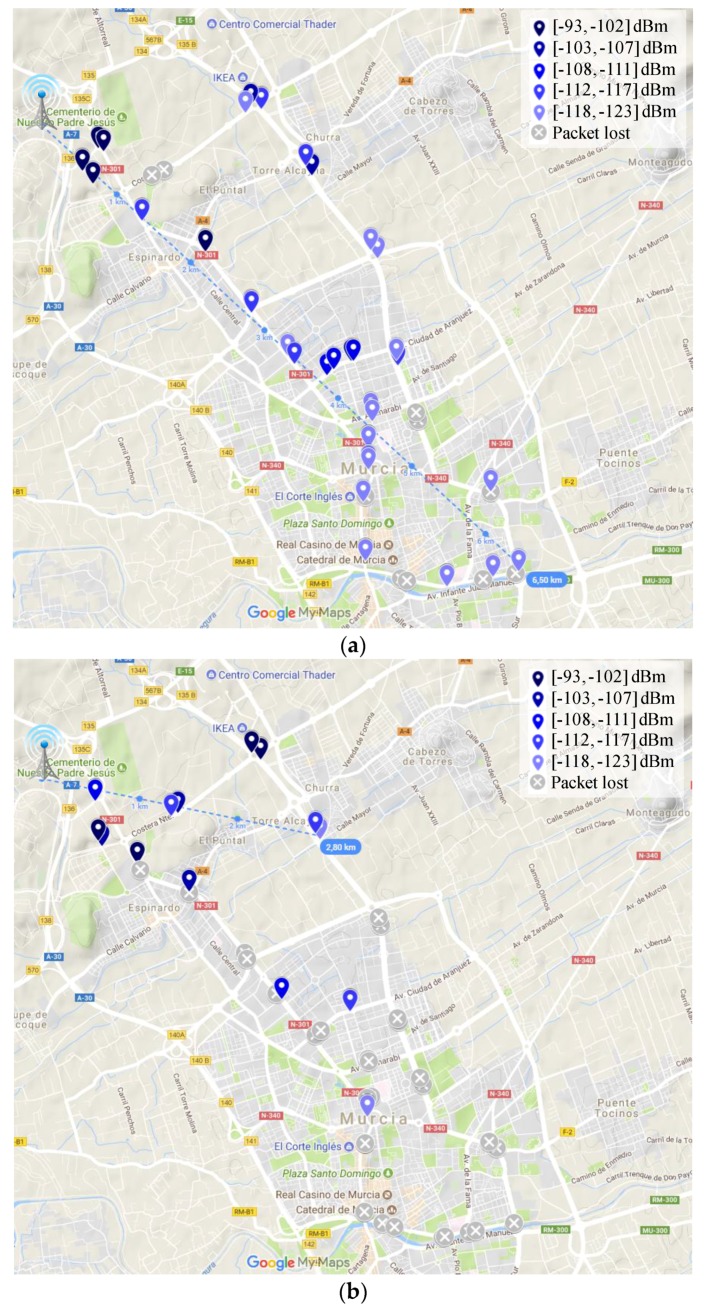
Received Signal Strength Indicator (RSSI) heat map in the urban scenario. (**a**) DR0; and (**b**) DR5.

**Figure 9:**
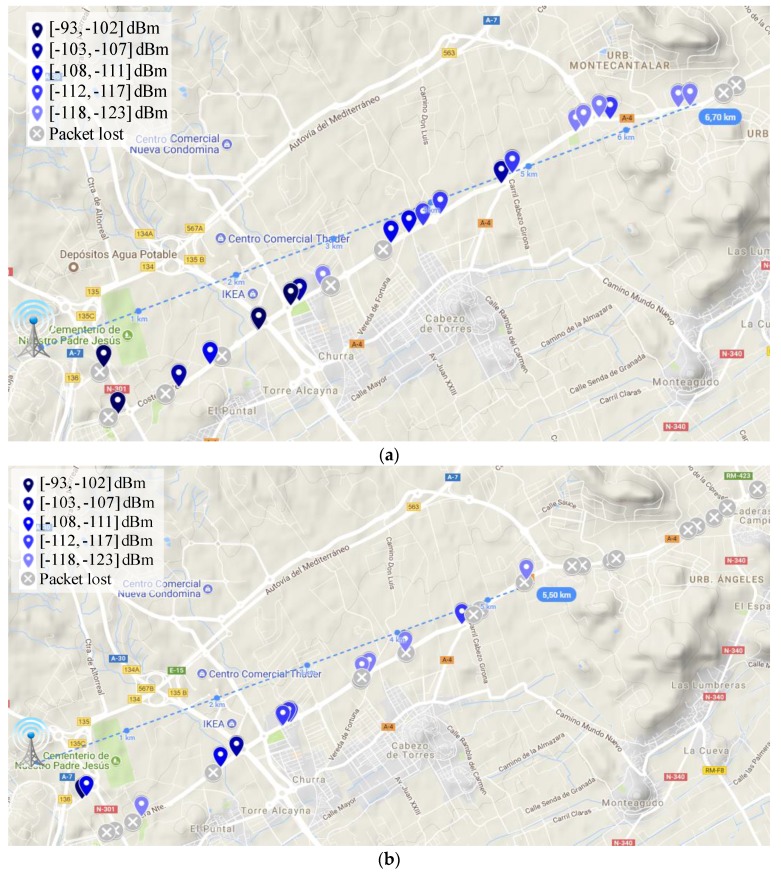
RSSI heat map in the suburban scenario. (**a**) DR0; and (**b**) DR5.

**Figure 10:**
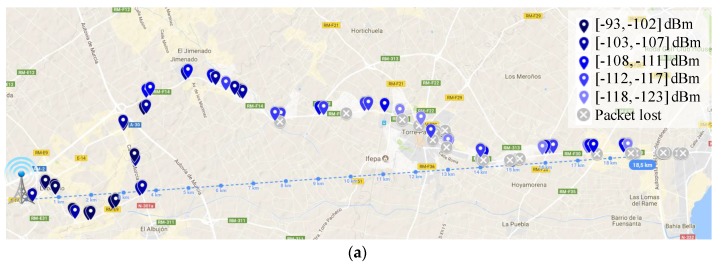

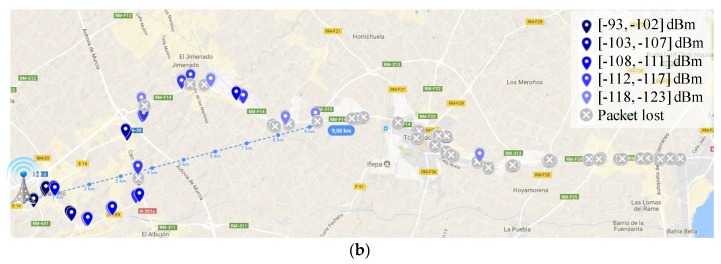
RSSI heat map in the rural scenario. (**a**) DR0; and (**b**) DR5.

After evaluating the studied scenarios from a theoretical perspective, in the following, the results obtained in our realistic sampling campaign are shown. Thereby, Figure 8, Figure 9 and Figure 10 depict the coverage range attained in each of the mentioned scenarios for the two extreme DR configurations, i.e., DR0 (SF = 12) and DR5 (SF = 7). In these RSSI heat maps, the possible different values for CR and packet length parameters are not distinguished; a discussion about the impact of these factors will be provided later on. First, observe the notable difference among the maximum link distances achieved in the three scenarios under consideration.

Whereas the maximum reached range is 6.5 km in urban conditions (DR0, Figure 8a), in the rural scenario, the furthest point with connectivity was attained at 18.5 km (DR0, Figure 10a). As expected, in between these results, the maximum range reached in the suburban scenario is 6.7 km (DR0, Figure 9a). Focusing on each individual scenario, note the impact of employing different DRs on the transmission range. For example, in the urban scenario, using a low DR (DR0) leads to an increase of the coverage of 132% (Figure 8a vs. Figure 8b). Note that in this scenario with DR5 (Figure 8b), there are longer transmissions that have not been taken as valid, as we consider them as lucky transmissions (please, observe the amount of lost transmission surrounding them). Table 4 presents the complete set of results regarding the longest link established in the three environments under study and considering the six different DR configurations. The obtained behavior is, as expected, that a higher DR leads to shorter covered distances. Some irregularities in this behavior, e.g., in the suburban scenario, are justified by the random presence of obstacles and the impact of vehicle mobility.

Regarding the validation of the theoretical study presented in the previous Section, observe that the distance covered by the base-station in each of the three environments under study is highly similar to that estimated by the radio planning tool. Particularly, the experimental results considering the lowest data-rate (DR0) closely match with the coverage areas presented previously. Please compare Figure 5 and Figure 8a, Figure 6 and Figure 9a, and Figure 7 and Figure 10a. In the light of these results, the consistency of both studies seems to be demonstrated, although the theoretical maps predict a slightly higher transmission range. This may be motivated by some deviations on the real sensitivity of the receivers or by the impact of small-scale obstacles that were not considered by the radio planning tool.

**Figure 11:**
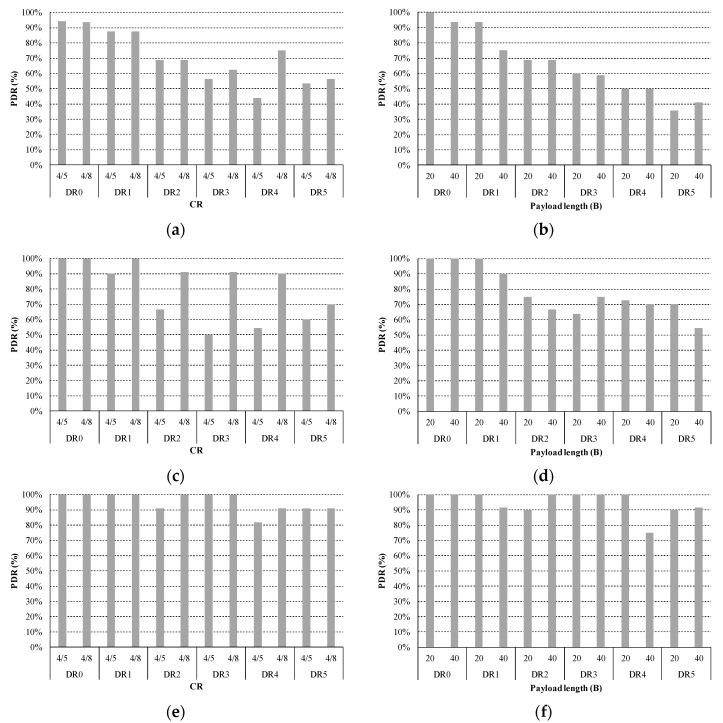
Impact of the CR and payload length on the PDR for the three evaluated scenarios. (**a**) Urban scenario, impact of CR; (**b**) Urban scenario, impact of payload length; (**c**) Suburban scenario. Impact of CR; (**d**) Suburban scenario, impact of payload length; (**e**) Rural scenario, impact of CR; (**f**) Rural scenario, impact of payload length.

With regard to the influence of LoRaWAN PHY layer settings on the system performance, in addition to the DR, the CR and payload length parameters were also examined during the experiments. Therefore, Figure 11 presents the level of PDR attained for the three studied scenarios when tuning both parameters. Note that, in order to elaborate these graphs, we have delimited the considered outcomes to those attained within a maximum range of 5 km from the base station. This facilitates result comparison by avoiding the bias introduced by excessively long transmissions that may artificially increment the level of packet loss in certain scenarios. Please, note that the longest range achieved in each scenario by each DR configuration is presented in Table 4. Focusing on the impact of the CR (Figure 11a,c,e), note that by adding extra redundant information in the LoRaWAN header (CR = 4/8), a noticeable improvement on the link reliability is obtained in most scenarios. This is especially notable for the case of the suburban scenario (Figure 11c). Observing this behavior, we can conclude that many of the transmissions were received partially corrupted and then rectified in the receiver by the forward error correction (FEC) mechanism. Thus, in this case, the extra overhead is justified in exchange of the increased link robustness provided by the FEC procedure. Regarding the payload length (Figure 11b,d,f), observe that even considering two short payload lengths, namely 20 B and 40 B, a perceptible PDR improvement is attained when employing the shortest one. This can be an expected result, as shorter transmissions are less prone to be impacted by the effect of interferences, collisions, and other typical harmful effects inherent to wireless systems.

In some tests, the attained values seem to be counterintuitive, e.g., in Figure 11b where the PDR for DR5 and payload 40 B is higher than the PDR obtained with a payload size of 20 B. These unusual values can be the result of the real traffic conditions experienced during the tests, like the speed at which the vehicle was circulating during transmission or the presence of sporadic obstacles between the base-station and the OBU. However, note that a clear trend is observed in the attained results.

**Figure 12:**
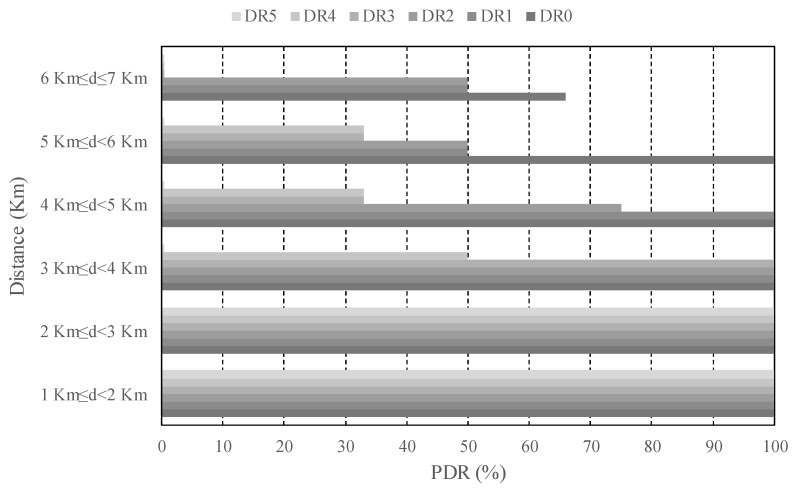
PDR evolution with the distance for each DR in the urban scenario. CR = 4/8, payload = 20 B.

In the light of these outcomes, it could be stated that the best option is always employing a low DR, e.g., DR0, and high CR, e.g., 4/8, for ensuring long connectivity and link reliability. However, there is an important trade-off related to the time-on-air (ToA) of each transmitted packet motivated by the low bit-rates available in LoRaWAN, which cause that the channel occupancy of each transmission was not negligible. In order to illustrate this assertion, Table 4 shows the theoretical ToA for the different configurations considered in our experiments. As discussed in [21], the restricted duty cycle in the ISM bands together with the long LoRaWAN ToA severely limits both the number of nodes than can simultaneously connect to a single base-station and the number of transmissions per node per day. For that reason, it is highly desirable to keep the ToA as short as possible, hence we argue that, depending on the distance of the end-node from the base-station, different DR should be employed with the aim of balancing transmission reliability and channel occupancy. In order to exemplify this strategy, Figure 12 depicts the PDR attained for each DR, considering the end-node distance to the base-station in the urban environment. This study is done for the most favorable configuration, i.e., CR = 4/8 and payload of 20 B. Thus, observe that it is not necessary to employ the lowest DR in distances below 3 km, as a PDR of 100% is attained for the highest DR. This leads to a reduction of 96% of the ToA of each transmission, from 2499 ms down to 103 ms (please, see Table 5). Note that the greatest DR (DR5) would be only recommendable in the furthest links of above 5 km. With this strategy, the reliability of the system can be maintained by selecting low DRs in adverse transmission conditions. At the same time, the channel occupancy is controlled by making use of high DRs when the transmission conditions are favorable. Overall, both consequences permit to improve the system scalability [21].

**Figure 13:**
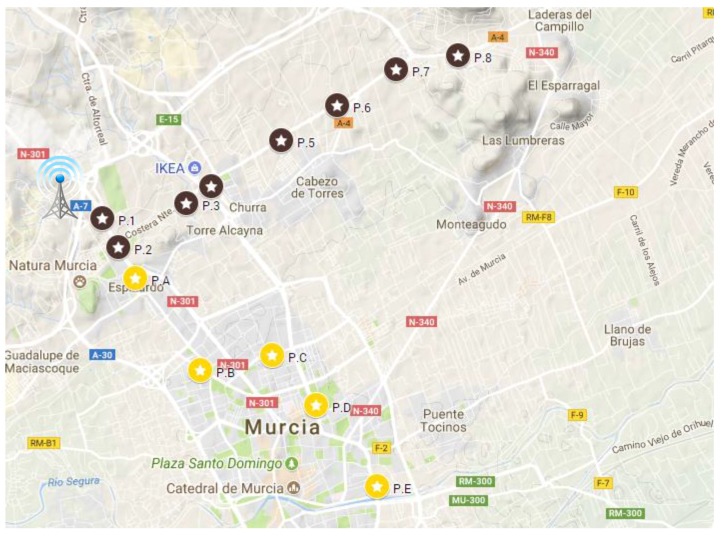
Sampling points for the nomadic test.

**Figure 14:**
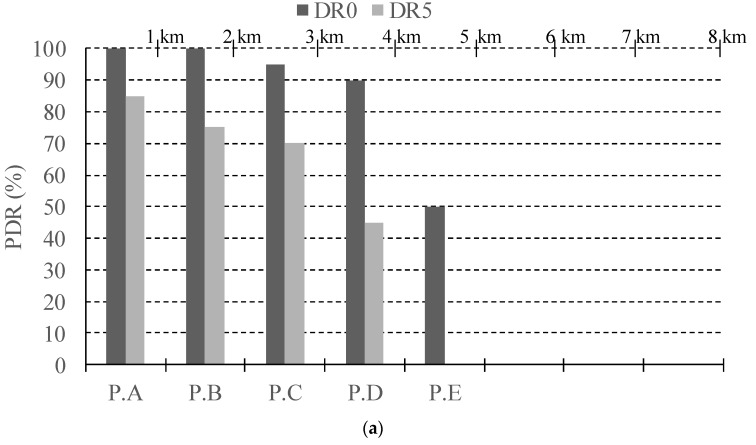

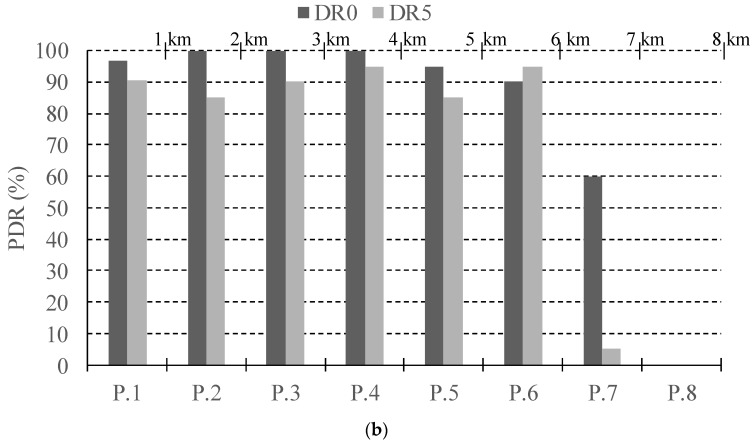
PDR obtained in the nomadic test for the urban and suburban scenarios with DR0 and DR5 for CR = 4/8, and payload length = 40 Bñ (**a**) Urban scenario; (**b**) Suburban scenario.

Previous works in the related literature has demonstrated that the vehicle motion can negatively impact on the reliability level of LoRaWAN transmissions [19]. For that reason, a nomadic test has also been conducted in the same urban and suburban scenarios in order to verify these observations, compare them with the results obtained under mobility conditions, and quantify the negative effect. A nomadic test consists in conducting a sampling campaign in specific points, i.e., with the car being stopped instead of doing it in motion. The selected sampling points for this experiment are shown in Figure 13. As in the previous experiment, different DR values have been studied; in this case, the CR was fixed to 4/8 and the payload length to 40 B. For the sake of simplicity and for permitting a direct comparison with the coverage results shown in Figure 8 and Figure 9, we focus on discussing the results attained for the two extreme DRs, i.e., DR0 and DR5. Thus, Figure 14 depicts the attained PDR in the different sampling points for the urban (Figure 14a) and suburban (Figure 14b) scenarios. With the aim of enabling the comparison between both scenarios and to provide information about the separation between the sampling points and the base station, a distance axis has been included also in those figures. Observe that considering the most conservative DR (DR0), the results were similar to the outcomes obtained under mobility conditions. In the urban scenario (Figure 14a), only the furthest sampling point, P.E, presented an excessive level of packet loss. The rest of sampling points did not seem to be influenced by the vehicle motion as the results in both tests were similar. The same behavior was observed in the suburban scenario (Figure 14b), with similar maximum link distances in both cases. Note that the sampling point P.7 was in the range limit obtained in the previous test (Figure 9a), which justifies the low level of PDR obtained there, and P.8 was out-of-range in both experiments, so no packets were received in any case. However, the results attained for the highest DR (DR5) slightly differed from one experiment to the other. The nomadic test in the urban scenario (Figure 14a) permitted a longer link range in comparison with the dynamic one (Figure 8b). This was evidenced by observing the sampling points P.C and, especially, P.D, which were out of range in the dynamic test but presented some level of received packets under static conditions. Additionally, in the suburban scenario the results in the sampling points P.5 and P.6 (Figure 14b) also improved those obtained under dynamic conditions (Figure 9b). By analyzing these results, it can be observed that LoRaWAN presents robustness to mobility, and consequently to Doppler effect, when using a low DR, i.e., when employing high values for the SF. However, by making use of higher DRs, end-node mobility emerges as an important element to be considered in order to protect the system reliability. As discussed previously, we face again a trade-off between system robustness and transmission rate, so the mobility characteristics of the scenario under consideration should be added to the afore-discussed environmental conditions when planning and deploying a large-scale LoRaWAN network. Therefore, prior to the network deployment phase, it is necessary to evaluate the terrain and scenario conditions aiming at predicting the power received by the end-nodes. To sum up, it can be concluded that LoRaWAN is a technology that presents reach features for providing long connectivity to IoT deployments in different scenarios with diverse propagation conditions. The election of the different LoRaWAN PHY layer configurations is determined by the trade-off between the link robustness and the transmission data-rate. Therefore, depending on the wireless link conditions and the end-node mobility, the system should adopt a more or less conservative configuration.

## 6. Conclusions

In this work, a comprehensive performance evaluation of LoRaWAN in several realistic scenarios has been presented. Different configurations of LoRaWAN PHY layer were explored in order to evaluate the most proper one depending on the propagation conditions. Three typical environments, namely, urban, suburban, and rural have been studied with the aim of considering their adversity to wireless transmissions and, consequently, their impact on the system performance. First, the estimated signal level in these scenarios have been evaluated by means of a precise planning tool, which employs topographic maps and the Okumura-Hata model. Thereafter, the attained outcomes from the theoretical study have been validated by an extensive sampling campaign. In the most adverse scenarios, i.e., urban and suburban, coverage ranges around 6 km were attained; in turn, in the open scenario (rural), a long transmission distance of over 18 km with the lowest data-rate was achieved. From the discussed results, it can be concluded that there is a clear trade-off between link robustness and data-rate (and consequently packet time-on-air); hence, depending on the propagation conditions and the distance between the base-station and the end-node, the LoRaWAN configuration parameters should be accordingly tuned. Particularly, in the urban scenario, for links shorter than 3 km, same levels of PDR were attained for the different DRs under evaluation. Therefore, with the aim of reducing the time-on-air for each packet, it is more convenient to employ high DRs. When the distance between both communication end-points is enlarged, lower DRs allow increasing the link robustness at the expense of dramatically decreasing the transmission rate. Finally, after evaluating the performance of the system under dynamic conditions, the interest was focused on examining the same scenarios but in a static situation to quantify the effect of mobility on performance. For that reason, a nomadic test was also conducted in order compare both scenarios. The results of this experiment confirmed that LoRaWAN presents a notable vulnerability related to the Doppler effect when using high transmission data-rates, but this effect is much less notable in the case of using low DRs. In the light of these results, it can be concluded that LoRaWAN presents a high level of adaptability to be employed in several IoT applications, such as smart cities, fleet/goods tracking, etc. Therefore, by applying the most proper configuration, the transmission system is able to establish long links with high robustness and low power consumption even under mobility conditions. For that reason, as future work, we plan to provide more intelligence to the system, in order to make it capable of auto-adapting the LoRaWAN PHY layer settings to current environmental conditions.

## Figures and Tables

**Table 1:** Strategy comparison among cellular communications, Wireless Sensor Networks (WSN), and Internet of Things (IoT) systems.

Cellular Communications	WSN	IoT
High bandwidth	Limited bandwidth	Limited bandwidth
Continuous traffic	Sporadic traffic	Sporadic traffic
Limited number of devices	High number of devices	Huge number of devices
Small area cells	Very small coverage	Wide area cells
Expensive network and devices	Reduced network and devices costs	Reduced network and device costs

**Table 2:** Summary of the equipment configuration for a BW of 125 kHz.

	End-Device	Base-Station
Output Power	+14 dBm	+14 dBm
Antenna Gain	+5 dBi	+8 dBi
Central frequency	868 MHz	868 MHz
RX Sensitivity SF = 7	−123.0 dBm	−126.5 dBm
RX Sensitivity SF = 8	−126.0 dBm	−129.0 dBm
RX Sensitivity SF = 9	−129.0 dBm	−131.5 dBm
RX Sensitivity SF = 10	−132.0 dBm	−134.0 dBm
RX Sensitivity SF = 11	−133.0 dBm	−136.5 dBm
RX Sensitivity SF = 12	−136.0 dBm	−139.5 dBm

**Table 3:** Vehicle speeds during the sampling tests.

Scenario	Average Speed (km/h) (Conf. Interval, α = 0.05)	Min. Speed (km/h)	Max. Speed (km/h)
Urban	22.3 (1.9)	0	53.5
Suburban	32.6 (2.3)	0	57.2
Rural	32.5 (1.5)	0	70.2

**Table 4:** Longest range (km) achieved in each scenario by each DR configuration.

DR	Urban Scenario	Suburban Scenario	Rural Scenario
DR0	6.5	6.7	18.5
DR1	5.4	5.8	13.7
DR2	4.5	5.7	11.6
DR3	4.4	5.3	11.5
DR4	4	5.0	10.3
DR5	2.8	5.5	9.6

**Table 5:** Theoretical time-on-air (ms) for the different tested LoRaWAN PHY layer configurations.

DR	Payload = 20 Bytes CR = 4/5|CR = 4/8	Payload = 40 Bytes CR = 4/5|CR = 4/8
DR0	1810|2499	2466|3547
DR1	987|1380	1315|1905
DR2	453|625	616|887
DR3	247|345	329|476
DR4	134|189	185|271
DR5	72|103	103|152
